# Optimizing the integration of modern systemic therapies and advanced radiotherapy techniques in breast cancer management: An expert opinion from the Institut Curie Breast Radiotherapy Group

**DOI:** 10.1002/ijc.70190

**Published:** 2025-10-11

**Authors:** Cezara Cheptea, Pierre Loap, Sofiane Allali, Alain Fourquet, Kim Cao, Youlia Kirova

**Affiliations:** ^1^ Institut Curie Breast Radiotherapy Group, Department of Radiation Oncology Institut Curie Paris France; ^2^ Laboratoire d'Imagerie Translationnelle en Oncologie (LITO) Institut Curie Orsay France; ^3^ Proton Therapy Center (CPO) Institut Curie Orsay France

**Keywords:** breast cancer radiotherapy, combined treatment, efficacy, systemic therapies, toxicity

## Abstract

The integration of modern systemic therapies with radiotherapy (RT) represents a promising strategy in breast cancer management, enhancing both locoregional control and systemic disease outcomes. This expert consensus from the Institut Curie Breast Radiotherapy Group focuses exclusively on modern systemic agents, synthesizing current evidence on the concurrent administration of human epidermal growth factor receptor 2‐targeted agents (trastuzumab, pertuzumab, trastuzumab emtansine, trastuzumab deruxtecan), cyclin‐dependent kinase 4 and 6 inhibitors (palbociclib, ribociclib), immunotherapies (pembrolizumab), poly(ADP‐ribose) polymerase inhibitors (olaparib), and new antibody‐drug conjugates (sacituzumab govitecan). Drawing from extensive clinical experience, including retrospective and prospective studies conducted at Institut Curie, this review provides a comprehensive analysis of the feasibility and safety of these novel combinations, ensuring an evidence‐based approach to optimizing breast cancer treatment strategies. In addition to systemic therapy considerations, this review highlights the importance of advanced RT techniques, including proton therapy, isocentric lateral decubitus positioning, and volumetric modulated arc therapy with deep inspiration breath hold, which play a crucial role in minimizing cardiac and pulmonary toxicities, particularly in patients receiving cardiotoxic agents or those with predisposing risk factors. By integrating both systemic advancements and optimized radiation delivery, this review provides a practical framework for the safe and effective combination of modern breast cancer therapies.

## Graphical abstract

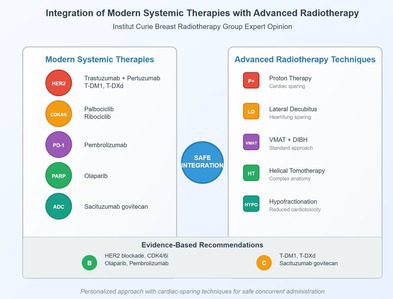

**Abbreviations**
CDK4/6i: cyclin‐dependent kinase 4 and 6 inhibitors
CNS: central nervous system
DIBH: deep inspiration breath hold
EFS: event‐free survival
EGFR: epidermal growth factor receptor
HER2: human epidermal growth factor receptor 2
HR+: hormone receptor positive
HT: helical tomotherapy
IC‐BRG: Institut Curie Breast Radiotherapy Group
IMRT: intensity‐modulated radiation therapy
LVEF: left ventricular ejection fraction
OS: overall survival
PARP: poly(ADP‐ribose) polymerase
PFS: progression‐free survival
RT: radiotherapy
SRS: stereotactic radiosurgery
T‐DM1: trastuzumab emtansine
T‐DXd: trastuzumab deruxtecan
TNBC: triple‐negative breast cancer
TROP2: trophoblast cell surface antigen 2
VMAT: volumetric modulated arc therapy
WBRT: whole‐brain radiotherapy

## 1 INTRODUCTION

Breast cancer management has undergone a profound transformation in recent years, marked by the advent of innovative systemic therapies that have redefined treatment paradigms and significantly improved patient outcomes. Modern therapeutic strategies now incorporate highly targeted agents, including hormone therapy, human epidermal growth factor receptor 2 (HER2)‐directed treatments, cyclin‐dependent kinase 4 and 6 inhibitors (CDK4/6i), immunotherapies, poly(ADP‐ribose) polymerase (PARP) inhibitors, and antibody–drug conjugates (e.g., trastuzumab deruxtecan [T‐DXd] and sacituzumab govitecan). These advancements enable a more personalized approach, tailoring treatment to specific molecular subtypes and optimizing therapeutic efficacy.

As systemic therapies become increasingly sophisticated, their concurrent integration with radiotherapy (RT) represents a promising avenue to further enhance tumor control. However, this approach introduces challenges regarding efficacy, toxicity, and optimal sequencing, necessitating a comprehensive evaluation of safety profiles and potential synergistic effects. The interaction between novel systemic agents and RT remains an area of active investigation, with existing clinical data often limited by heterogeneity in RT techniques, dosing strategies, and fractionation schedules. Furthermore, the majority of available evidence is derived from retrospective analyses or early‐phase studies, emphasizing the need for rigorous prospective validation.

Beyond systemic considerations, advancements in RT techniques play a critical role in safely integrating modern systemic therapies while minimizing toxicities. Proton therapy, an emerging modality, offers significant dosimetric advantages by sparing cardiac and pulmonary structures, making it particularly relevant for patients receiving cardiotoxic agents such as HER2‐targeted therapies or immune checkpoint inhibitors.^1^, ^2^, ^3^, ^4^, ^5^ Isocentric lateral decubitus positioning has also been shown to effectively reduce radiation dose to the heart and lungs, offering a simple and widely implementable alternative to traditional supine positioning.^6^, ^7^, ^8^ In the absence of these techniques, volumetric modulated arc therapy (VMAT) with deep inspiration breath hold (DIBH) should be considered the standard approach, particularly for patients requiring locoregional irradiation.^9^, ^10^ Additionally, helical tomotherapy (HT) has demonstrated excellent long‐term outcomes with minimal late toxicity, particularly in complex anatomical cases.^11^

This literature review presents an expert opinion from the Institut Curie Breast Radiotherapy Group (IC‐BRG) on the integration of modern systemic therapies—including anti‐HER2 and trophoblast cell surface antigen 2 (TROP2) antibody–drug conjugates, PARP inhibitors, CDK4/6i, and immunotherapies—with RT in breast cancer management. These recommendations are grounded in the extensive experience of the IC‐BRG, drawing from retrospective and prospective data collected within the group. By synthesizing the latest evidence and leveraging institutional expertise, this review aims to provide practical guidance for optimizing treatment strategies, incorporating both systemic and technical innovations to ensure the safe and effective combination of cutting‐edge systemic agents with RT.

## 2 METHODOLOGY

This expert opinion represents a narrative review of available evidence combined with institutional experience from the IC‐BRG. A comprehensive literature search was conducted using the PubMed database to identify studies evaluating the concurrent use of modern systemic therapies with RT in breast cancer patients. The search strategy included combinations of the following terms: “breast cancer,” “radiotherapy,” “radiation therapy,” combined with specific systemic therapy terms including “trastuzumab,” “pertuzumab,” “T‐DM1,” “trastuzumab deruxtecan,” “T‐DXd,” “palbociclib,” “ribociclib,” “CDK4/6 inhibitors,” “pembrolizumab,” “immunotherapy,” “olaparib,” “PARP inhibitors,” and “sacituzumab govitecan.” Studies were included if they reported concurrent use of modern systemic therapies with RT in breast cancer populations, included safety and/or efficacy outcomes, and consisted of case series with five or more patients. Case reports with fewer than five patients, preclinical studies, and non‐breast cancer populations were excluded. Literature published between January 2010 and March 2025 was reviewed, with only English‐language publications included. Evidence quality was assessed and recommendations were formulated based on available evidence strength combined with institutional clinical experience. Where high‐quality evidence was limited, recommendations reflect expert opinion based on institutional experience and extrapolation from related studies.

To provide transparent guidance on the strength of evidence supporting our recommendations, we have systematically evaluated each treatment combination using an established evidence classification framework. Table 1 summarizes the evidence level and recommendation grade for each concurrent systemic therapy and RT combination discussed in this expert opinion. Evidence levels range from Level I representing high‐quality randomized controlled trials and meta‐analyses to Level IV encompassing expert opinion and case reports. Recommendation grades range from Grade A representing strong recommendations based on high‐quality evidence to Grade D indicating insufficient evidence to make a recommendation.

**TABLE 1:** Evidence summary for concurrent systemic therapy and radiotherapy combinations.

Treatment combination	Evidence level	Recommendation grade	Rationale
Dual HER2 blockade (trastuzumab + pertuzumab) with radiotherapy	III	B (moderate)	Consistent safety data across multiple retrospective studies
T‐DM1 with radiotherapy	III	C (weak)	Limited data with acceptable preliminary safety profile
T‐DXd with radiotherapy	III	C (weak)	Emerging evidence with short follow‐up
CDK4/6i with radiotherapy	III	B (moderate)	Multiple consistent retrospective series
Pembrolizumab with radiotherapy	II–III	B (moderate)	Phase 2 trial data supported by institutional experience
Olaparib with radiotherapy	II	B (moderate)	Dedicated prospective dose‐escalation study
Sacituzumab govitecan with radiotherapy	III	C (weak)	Very limited preliminary data

Abbreviations: CDK4/6i, cyclin‐dependent kinase 4 and 6 inhibitors; HER2, human epidermal growth factor receptor 2; T‐DM1, trastuzumab emtansine; T‐DXd, trastuzumab deruxtecan.

Evidence level classification:

- Level I: High‐quality randomized controlled trials, systematic reviews, meta‐analyses.
- Level II: Well‐designed prospective cohort studies, large retrospective cohort studies.
- Level III: Case series, small retrospective studies, institutional experiences.
- Level IV: Expert opinion, case reports.

Recommendation grade classification:

- Grade A: Strong recommendation based on high‐quality evidence (high confidence).
- Grade B: Moderate recommendation based on moderate‐quality evidence (moderate confidence).
- Grade C: Weak recommendation based on low‐quality evidence or expert opinion (low confidence).
- Grade D: Insufficient evidence to make a recommendation.

## 3 DUAL HER2 BLOCKADE (TRASTUZUMAB AND PERTUZUMAB)

Trastuzumab, a monoclonal antibody targeting the extracellular domain of the HER2 receptor, exerts its antitumor effects by inhibiting the MAP kinase and PI3K‐Akt signaling pathways, thereby reducing tumor proliferation and enhancing immune‐mediated tumor cell destruction. Pertuzumab, another HER2‐targeted monoclonal antibody, binds the dimerization domain of HER2, blocking its interaction with EGFR, HER3, and HER4. This dual HER2 blockade enhances tumor suppression and has demonstrated improved clinical outcomes across various stages of HER2‐positive breast cancer. Given the increasing use of dual HER2‐targeted therapy, its concurrent administration with RT has been the focus of multiple retrospective studies at Institut Curie, progressively evaluating the safety of this approach over time.

The first study evaluating this combination at Institut Curie was conducted by Castel‐Ajgal et al.,^12^ who assessed 23 patients receiving RT while on maintenance trastuzumab and pertuzumab after completing chemotherapy. The study included chest irradiation (50 Gy in 25 fractions) with nodal RT in most cases, while five patients received palliative RT. With a median follow‐up of 12.6 months, the treatment was well tolerated. No symptomatic cardiac events were reported, though one patient experienced an asymptomatic decrease in left ventricular ejection fraction (LVEF) below 50%. Acute toxicities primarily consisted of radiodermatitis, with one patient experiencing Grade 3 toxicity, six with Grade 2, and five with Grade 1. Additionally, two cases of Grade 2 esophagitis and two cases of asymptomatic Grade 1 radiation pneumonitis were observed. This study provided initial evidence suggesting that the combination of trastuzumab, pertuzumab, and RT was feasible with manageable toxicity. Building upon these findings, Ben Dhia et al.^13^ conducted a larger retrospective study including 77 patients treated with concurrent RT, trastuzumab, and pertuzumab. The study population consisted of patients with metastatic (64.9%) or unresectable recurrent disease (35.1%). RT fields included whole breast irradiation (53.2%), chest wall irradiation (37.7%), and regional lymph node irradiation (68.8%). Palliative RT was administered in 26% of cases, with bone irradiation in 15.6% of patients and brain irradiation in 9.2%. Acute toxicities were primarily dermatologic, with 46.8% of patients experiencing Grade 1 radiodermatitis, 22.1% experiencing Grade 2, and 3.9% experiencing Grade 3. One patient developed Grade 2 esophagitis, and 7.7% experienced an asymptomatic decline in LVEF. No cases of radiation‐induced pneumonitis were reported. Late toxicity was infrequent, with only one case of Grade 3 cardiac toxicity occurring 8 months after concurrent treatment. These results further reinforced the safety of trastuzumab and pertuzumab administration during RT, demonstrating that even with longer follow‐up, significant toxicity remained rare. A subsequent study by Aboudaram et al.^14^ provided an even more refined analysis by focusing exclusively on patients receiving curative‐intent RT in the setting of HER2‐positive breast cancer, excluding those receiving palliative irradiation. This study included 55 patients treated between 2013 and 2019 with a median follow‐up of 4.1 years. All patients received dual HER2 blockade with trastuzumab and pertuzumab alongside curative dose RT (median dose: 50 Gy). The safety evaluation confirmed the excellent tolerance of this combination, with only three cases of Grade 3 radiodermatitis (5.4%) and no significant gastrointestinal or cardiac toxicities. The mean decrease in LVEF before chemotherapy and after RT was statistically significant (−2.43%, *p* < .01) but remained clinically minor. Notably, locoregional control and survival outcomes were comparable to those of HER2‐positive breast cancer patients receiving dual HER2 blockade without concurrent RT.

### 3.1 Recommendations for clinical practice

The accumulated evidence from these three successive studies at Institut Curie strongly supports the feasibility and safety of concurrent trastuzumab, pertuzumab, and RT in HER2‐positive breast cancer. Across progressively larger patient cohorts and longer follow‐ups, no major increases in RT‐related toxicity have been observed. Reported toxicities, including radiodermatitis and esophagitis, remain within expected ranges and do not appear exacerbated by dual HER2 blockade. Importantly, cardiac tolerance has been reassuring, with only mild and mostly asymptomatic reductions in LVEF. Cardiovascular risk factors should still be carefully assessed, and routine cardiac monitoring before, during, and after treatment is recommended. While retrospective data are reassuring, further prospective trials are warranted to refine long‐term cardiac safety assessments and optimize treatment sequencing.

## 4 TRASTUZUMAB EMTANSINE

Trastuzumab emtansine (T‐DM1), an antibody–drug conjugate, combines trastuzumab with the cytotoxic agent maytansine and is particularly effective in HER2‐positive breast cancer patients with residual invasive disease following neoadjuvant therapy. By binding to HER2 receptors, T‐DM1 facilitates targeted intracellular delivery of the cytotoxic DM1, leading to cell cycle arrest and apoptosis (Geraud). Given the growing use of T‐DM1 in the adjuvant setting, its safety profile when combined with RT has become a subject of increasing clinical interest.

Retrospective and early prospective studies have assessed the feasibility of concurrent T‐DM1 and RT, reporting generally favorable tolerability with mild‐to‐moderate acute toxicities. A study by Zolcsak et al.^15^ at Institut Curie evaluated 14 patients treated with concurrent T‐DM1 and RT for non‐metastatic HER2‐positive breast cancer with residual invasive disease. Patients received a total RT dose of 50 Gy to the breast or chest wall, with nodal irradiation in 10 cases and a tumor bed boost in 4 cases. The most frequently observed toxicity was Grade 1 radiodermatitis, while two patients experienced a reversible Grade 2 decrease in LVEF. Additionally, transient alanine aminotransferase elevations were noted in three patients, with one case each of Grade 1, Grade 2, and Grade 3 toxicity. The study concluded that acute skin and cardiac toxicities remained within acceptable limits, supporting the feasibility of concurrent T‐DM1 and RT but highlighting the need for further research.

Regarding central nervous system (CNS) irradiation, a study by Geraud et al.^16^ found that while T‐DM1 combined with stereotactic radiosurgery (SRS) for brain metastases was generally well tolerated, an increased incidence of radiation necrosis (6%–11%) was observed. This finding suggests that careful patient selection and dose optimization may be necessary when considering concurrent T‐DM1 and brain‐directed RT. Additionally, while no significant increase in acute or late toxicities has been consistently reported, concerns regarding radiation‐induced cardiac injury warrant long‐term follow‐up to better characterize late‐onset toxicities.

### 4.1 Recommendations for clinical practice

Current data suggest that concurrent T‐DM1 and RT is feasible with an acceptable toxicity profile, particularly in non‐metastatic HER2‐positive breast cancer patients requiring locoregional RT. However, cardiac monitoring remains crucial, as mild but reversible decreases in LVEF have been reported. For patients receiving CNS irradiation, caution is advised due to the potential risk of increased radiation necrosis.

## 5 TRASTUZUMAB DERUXTECAN

The DESTINY‐Breast03 trial demonstrated a significant improvement in progression‐free survival (PFS) with T‐DXd compared to T‐DM1 (28.8 months vs. 6.8 months), while the DESTINY‐Breast04 study^17^ confirmed its efficacy in HER2‐low metastatic breast cancer, showing superior survival outcomes over conventional chemotherapy. Given the increasing use of T‐DXd in HER2‐positive and HER2‐low metastatic breast cancer, its combination with RT has raised safety concerns, particularly regarding potential toxicities.

Recent retrospective analyses have provided insights into the safety profile of concurrent T‐DXd and RT. A study by Bouziane et al.^18^ at Institut Curie evaluated 33 patients with HER2‐positive and HER2‐low metastatic breast cancer who underwent RT while receiving T‐DXd. With a median follow‐up of 11 months, the most common acute toxicity was nausea (Grade 1), while 21.2% of patients developed Grade 2 toxicities, including asthenia, mucositis, cardiac decompensation, and diarrhea. Late toxicities were reported in 21.2% of patients, with nausea being the most frequent. Systemic therapy discontinuation occurred in five patients due to nausea, thrombocytopenia, neutropenia, or cardiac decompensation, although no RT‐specific discontinuation was noted. The study concluded that concurrent T‐DXd and RT appears to be feasible, with an acceptable toxicity profile. Further supporting these findings, the TENDANCE study,^19^ the largest retrospective multicenter analysis to date, examined 54 patients treated with concurrent T‐DXd and RT. The cohort included HER2‐positive (40.7%) and HER2‐low patients (59.3%), with T‐DXd administered as second‐line (18.2%), third‐line (31.8%), or later‐line therapy (50%). RT was delivered with either palliative (72.2%) or ablative intent (27.8%), primarily targeting bone metastases (46.3%) and brain lesions via SRS (25.9%). At a median follow‐up of 9 months, 22.2% of patients achieved a complete response, while 77.8% had partial response or stable disease. Grade 1 or 2 asthenia was reported in 51.8% of patients, while only 16.6% experienced other Grade 1 or 2 adverse events. Importantly, no T‐DXd therapy discontinuation was directly related to RT.

Collectively, these findings suggest that concurrent administration of T‐DXd and RT is feasible, with a manageable toxicity profile. While pulmonary toxicity remains a theoretical concern, as interstitial lung disease or pneumonitis has been reported in 12.1% of patients receiving T‐DXd,^17^ current data do not indicate a significant increase in radiation‐induced pulmonary or cardiac toxicity. For CNS irradiation, preliminary results suggest that combining T‐DXd with SRS or whole‐brain RT (WBRT) does not substantially elevate toxicity risk. However, careful patient selection and advanced RT planning remain essential, particularly for thoracic irradiation, where minimizing lung exposure may help mitigate potential risks.

### 5.1 Recommendations for Clinical Practice

Concurrent T‐DXd and RT appears to be a viable approach with an acceptable safety profile. Given the manageable toxicity rates reported, this combination may be considered when RT is clinically indicated, particularly for brain and bone metastases. However, caution should be exercised in patients at risk of pulmonary or cardiac complications, and RT planning should aim to minimize organ exposure. Future prospective studies with longer follow‐up are needed to confirm these findings, establish optimal treatment sequencing, and refine patient selection criteria to optimize both safety and therapeutic benefit.

## 6 PALBOCICLIB AND RIBOCICLIB

CDK4/6i, including palbociclib, ribociclib, and abemaciclib, have become a cornerstone in the treatment of hormone receptor‐positive (HR+), HER2‐negative locally advanced or metastatic breast cancer. Administered alongside endocrine therapy, such as aromatase inhibitors or fulvestrant, CDK4/6i have demonstrated significant clinical benefits. In premenopausal patients, their use necessitates concurrent ovarian suppression. While these agents have an established safety profile, their concomitant use with RT remains an area of clinical uncertainty, particularly when RT is indicated for locoregional disease control or symptomatic metastases.

Recent retrospective analyses have explored the safety and feasibility of concurrent administration. A study by Beddok et al.^20^ at Institut Curie retrospectively evaluated 30 patients who received palbociclib concurrently with RT, including both locoregional and metastatic sites. Acute toxicities primarily included radiodermatitis and neutropenia, with palbociclib discontinuation required in three cases due to Grade 3 radiodermatitis, febrile neutropenia, or disease progression. Importantly, no late toxicities were observed at a minimum follow‐up of 6 months, suggesting that concurrent administration is feasible but requires careful monitoring. A subsequent retrospective study by Beddok et al.^21^ further examined the outcomes and toxicity profiles of patients receiving concurrent CDK4/6i and locoregional RT, including irradiation of the breast with a boost, the thoracic wall post‐mastectomy, and the regional lymph nodes. The study included 27 patients with de novo metastatic HR+/HER2− breast cancer receiving CDK4/6i as first‐line metastatic treatment. The median duration of concomitant RT and CDK4/6i administration was 21 days, with neutropenia (44%) and dermatitis (37%) being the most frequently observed acute toxicities. The severity of dermatitis correlated with larger treatment volumes (clinical target volume >911cc and planning target volume >1285cc). CDK4/6i were temporarily discontinued in five patients due to toxicity (*n* = 3) or disease progression (*n* = 2). One patient developed Grade 2 late pulmonary fibrosis. Despite these events, the study concluded that concurrent CDK4/6i therapy and RT did not result in severe late toxicity for the majority of patients, with 1‐ and 3‐year progression‐free survival rates of 61.4% and 53.7%, respectively.

The feasibility of combining ribociclib with RT was preliminarily explored by Bouziane et al.,^22^ which assessed 38 patients treated for metastatic HR+/HER2− breast cancer with ribociclib and palliative RT. Among them, 36 patients temporarily discontinued ribociclib, while two continued treatment concurrently with irradiation of bone metastases (20 Gy in five fractions). Both patients experienced pain relief, with no interruptions in RT. However, ribociclib was suspended in both cases—one due to Grade 3 neutropenia and the other due to Grade 1 QTc interval prolongation. One patient required a dose reduction to 400 mg, with favorable treatment outcomes. Notably, no late toxicities were observed, and both patients demonstrated disease control, with one achieving complete remission of bone metastases and the other partial remission.

### 6.1 Recommendations for clinical practice

Retrospective data consistently support the feasibility and acceptable safety of concurrently administering CDK4/6i with RT, including both palliative and locoregional treatments. Palbociclib and ribociclib have demonstrated particularly encouraging safety profiles, suggesting that clinicians can maintain CDK4/6i therapy during RT without significant additional risks. Large‐scale prospective studies, including the ongoing PALATINE trial, are needed to confirm these preliminary findings and establish definitive safety guidelines. Until further high‐quality evidence is available, concurrent CDK4/6i therapy with RT should be carefully considered on a case‐by‐case basis, with treatment decisions tailored to individual patient risk factors and therapeutic goals.

## 7 PEMBROLIZUMAB

Triple‐negative breast cancer (TNBC) is an aggressive subtype associated with a poor prognosis. The introduction of immune checkpoint inhibitors, particularly pembrolizumab (anti‐PD‐1), has significantly improved clinical outcomes by reversing immune suppression and enhancing the activity of tumor‐infiltrating lymphocytes within the tumor microenvironment. The KEYNOTE‐522 Phase 3 trial^23^ demonstrated the benefit of incorporating pembrolizumab into neoadjuvant and adjuvant therapy for locally advanced TNBC, increasing 3‐year event‐free survival (EFS) from 76.8% to 84.5%. Given the well‐established role of adjuvant RT in improving local control and breast cancer‐specific survival, the concurrent administration of pembrolizumab and RT represents a potentially synergistic strategy. RT has been shown to modulate the tumor microenvironment, potentially enhancing immune‐mediated tumor eradication. However, the safety and tolerability of this combination, particularly in the adjuvant setting, remain an area of clinical interest.

A recent bicentric ambispective study by Tison et al.^24^ evaluated the tolerance profile of concurrent adjuvant RT and pembrolizumab in patients with early and locally advanced TNBC. The study included 55 patients who had previously received neoadjuvant chemo‐immunotherapy with pembrolizumab. Among them, 28 patients received adjuvant RT concurrently with pembrolizumab (RT + P group), while 27 patients had pembrolizumab withheld during RT (RT‐only group). The results demonstrated no significant difference in toxicity between the two groups. Grade ≥3 adverse events were infrequent, with one case of Grade 3 pain in the RT + P group and one case of Grade 3 radiodermatitis in the RT‐only group. Importantly, no cardiac or pulmonary toxicity was reported during RT, and with a median follow‐up of 12 months, no patient experienced disease relapse.

These findings suggest that concurrent administration of pembrolizumab and adjuvant RT is well tolerated, with no apparent increase in acute toxicity. A Phase 2 study evaluating pembrolizumab with palliative RT in metastatic TNBC^25^ similarly reported a favorable safety profile, with only mild, manageable RT‐related skin toxicities. While preliminary data are reassuring, further studies with extended follow‐up are necessary to better assess the potential risk of late‐onset toxicities, particularly immune‐related myocarditis and pulmonary adverse events. Optimized RT techniques aimed at minimizing unintentional cardiac exposure may help mitigate potential risks in patients receiving concurrent pembrolizumab.

### 7.1 Recommendations for clinical practice

Preliminary data indicate that the combination of pembrolizumab and RT is a well‐tolerated and feasible approach in TNBC patients. However, further prospective studies are required to establish safety guidelines and better assess the risk of long‐term toxicities, particularly cardiac and pulmonary events. Notably, in patients with a history of pembrolizumab‐induced myocarditis, exposure to low‐dose radiation may potentially trigger the reactivation of autoreactive lymphocytic clones, warranting careful consideration in treatment planning.

## 8 OLAPARIB

PARP inhibitors, such as olaparib, leverage synthetic lethality to target TNBC, which frequently exhibits homologous recombination deficiencies. By inhibiting PARP‐mediated DNA repair, olaparib amplifies radiation‐induced DNA damage, potentially enhancing the therapeutic efficacy of RT in TNBC.^26^ The feasibility and safety of this approach were evaluated in the RADIOPARP Phase I trial, a prospective dose‐escalation study assessing concurrent olaparib with locoregional breast RT in patients with advanced, inflammatory, or residual TNBC following neoadjuvant chemotherapy.

Olaparib was escalated up to 200 mg twice daily without reaching a maximum tolerated dose or encountering dose‐limiting toxicities. At 1‐year follow‐up, adverse events remained mild, with only 4.2% of patients experiencing persistent Grade 2 toxicities, including breast pain, fibrosis, and cosmetic deformity.^27^ The long‐term safety profile remained favorable at 5 years, with no late grade ≥3 treatment‐related toxicities observed.^28^ Furthermore, 3‐year overall survival (OS) and EFS rates were reported at 83% and 65%, respectively.^29^

### 8.1 Recommendations for clinical practice

The 200 mg twice daily dose of olaparib is supported for use in clinical trials due to its demonstrated safety. Future studies may refine patient selection criteria and optimize treatment sequencing to maximize therapeutic benefit while minimizing toxicity.

## 9 SACITUZUMAB GOVITECAN

Sacituzumab govitecan, an anti‐TROP2 antibody‐drug conjugate, is an established treatment for metastatic TNBC and late‐line hormone receptor‐positive/HER2‐negative (HR+/HER2−) disease. In metastatic settings, RT is frequently required for symptom control. However, the feasibility of combining sacituzumab govitecan with RT remains unclear, as standard practice typically recommends discontinuing systemic treatments before irradiation to minimize toxicity risks. Given the aggressive nature of metastatic disease, prolonged treatment interruptions may compromise tumor control, raising the need to evaluate the safety of concurrent administration.

A retrospective, single‐center study assessed the safety of concurrent RT and sacituzumab govitecan in metastatic breast cancer patients.^30^ Thirteen patients received both treatments simultaneously, primarily for brain and bone metastases. With a median follow‐up of 5 months, no cases of RT‐induced toxicity were observed, including in patients who underwent whole‐brain irradiation. Sacituzumab govitecan was well tolerated, with no treatment interruptions required due to toxicity. Grades 3 and 4 adverse events were limited to neutropenia, consistent with the known safety profile of sacituzumab govitecan.

### 9.1 Recommendations for clinical practice

These findings suggest that concurrent sacituzumab govitecan and RT is feasible and well tolerated, particularly for brain and bone metastases. Clinicians may consider this combination when RT is required for symptom management, without necessitating prolonged treatment suspension. However, given the limited sample size and follow‐up, further prospective studies are needed to validate these findings and assess potential toxicity risks in other metastatic sites, such as thoracic or abdominal lesions.

## 10 OPTIMIZING RT TECHNIQUES TO MINIMIZE CARDIOTOXICITY IN THE CONTEXT OF MODERN SYSTEMIC THERAPIES

As modern systemic therapies—particularly HER2‐targeted agents (trastuzumab, pertuzumab, T‐DM1, T‐DXd), CDK4/6i, and pembrolizumab—are increasingly integrated with RT, the need for advanced RT delivery techniques to minimize cardiac and pulmonary toxicity has become a central concern. Many of these systemic agents have cardiotoxic potential, including left ventricular dysfunction, myocarditis, and increased susceptibility to radiation‐induced injury. Thus, optimizing RT techniques is crucial to ensuring the safety of concurrent treatments. The IC‐BRG has been a leader in implementing cardiac‐sparing RT strategies, including proton therapy, isocentric lateral decubitus positioning, and VMAT with DIBH.

Proton therapy offers significant dosimetric advantages by reducing radiation exposure to the heart, lungs, and contralateral breast. Breast proton therapy effectively limits cardiac dose,^1^, ^2^, ^31^ making it particularly relevant for patients receiving cardiotoxic systemic agents or those with a history of immune‐related myocarditis from pembrolizumab. Even low‐dose radiation exposure to the heart may exacerbate immune‐mediated cardiac inflammation, reinforcing the need for cardiac‐sparing strategies in this population.^32^, ^33^

For patients in whom proton therapy is not available, isocentric lateral decubitus positioning^6^ is a highly effective alternative to reduce radiation dose to the heart and lungs. A study by Loap et al.^8^ showed that this technique significantly reduces mean heart dose and dose to cardiac substructures (left ventricle, right and left coronary arteries, sinoatrial node^34^, ^35^, ^36^) compared to standard supine positioning. This method is particularly valuable for patients receiving HER2‐targeted therapies or immune checkpoint inhibitors, where minimizing cardiac radiation exposure is a priority.

When neither proton therapy nor isocentric lateral decubitus positioning is available, VMAT with DIBH should be considered the standard approach for cardiac sparing. While Loap et al. found that DIBH does not provide significant additional cardiac protection in right‐sided breast cancer,^10^ for left‐sided tumors or cases requiring nodal irradiation, it remains an effective option to reduce cardiac exposure.^37^ Additionally, HT has demonstrated excellent long‐term outcomes,^11^, ^38^ with no reported cardiac, pulmonary, thyroid, or digestive toxicities at 10 years, making it a viable approach for patients with complex anatomy requiring highly conformal radiation delivery.^11^ Finally, hypofractionation reduces biological effective dose to cardiac substructures and should be considered to further reduce the risk of long‐term radiation‐induced cardiotoxicity.^39^, ^40^

### 10.1 Recommendations for clinical practice

Given the growing integration of cardiotoxic systemic therapies with RT, the IC‐BRG recommends a stepwise approach to cardiac sparing, prioritizing:

1. Proton therapy for patients at highest risk, particularly those receiving cardiotoxic agents or with a history of pembrolizumab‐induced myocarditis.
2. Isocentric lateral decubitus positioning as an excellent alternative to reduce cardiac and pulmonary exposure, particularly for patients receiving HER2‐targeted therapies.
3. VMAT with DIBH as the standard technique when proton therapy or lateral decubitus is not available.
4. HT in patients with complex anatomy, ensuring homogeneous dose distribution with minimal toxicity.

As the IC‐BRG continues to refine breast cancer RT techniques, future research should focus on further optimizing patient selection criteria for each approach, expanding access to proton therapy, and conducting prospective studies to evaluate long‐term outcomes in the context of modern systemic therapies.

## 11 DISCUSSION

The integration of modern systemic therapies with RT represents an evolving area in breast cancer management, with evidence quality varying significantly across different treatment combinations. It is important to distinguish between recommendations supported by external validation versus those based primarily on institutional experience.

Limited high‐quality external evidence exists for most combinations. The KEYNOTE‐522 trial provides robust evidence for pembrolizumab^23^ in TNBC, while the RADIOPARP phase 1 trial offers prospective data for the olaparib combination.^29^ The DESTINY‐Breast trials establish T‐DXd efficacy^17^ but do not specifically address RT combinations. A substantial portion of the safety data derives from Institut Curie institutional studies.^12^, ^13^, ^14^, ^15^, ^18^, ^21^, ^22^, ^30^ While these studies provide valuable real‐world experience with large patient cohorts and extended follow‐up, they represent single‐institutional experience with potential selection bias and protocol standardization that may not reflect broader clinical practice. The TENDANCE multicenter study^19^ provides important external validation for T‐DXd combinations, while scattered case series from other institutions offer limited validation for other combinations. However, most external reports consist of small series with short follow‐up, limiting their ability to definitively establish safety profiles. Our strongest recommendations reflect combinations supported by either prospective trials such as those for olaparib and pembrolizumab, or multiple consistent institutional series with external corroboration including dual HER2 blockade and CDK4/6i. Weaker recommendations reflect combinations with limited external validation or very recent introduction into clinical practice. This distinction is crucial for clinicians when applying these recommendations, as institutional experience, while valuable, requires external validation before widespread adoption. The ongoing PALATINE trial (NCT03870919) for CDK4/6i and future prospective studies will be essential to validate these preliminary findings and establish evidence‐based guidelines for concurrent therapy administration.

For HER2‐positive breast cancer, data support the concurrent use of trastuzumab and pertuzumab with RT, demonstrating manageable toxicity with no major increase in cardiac events. The experience at Curie, with successive evaluations from Castel‐Ajgal et al.,^12^ Ben Dhia et al.,^13^ and Aboudaram et al.,^14^ reinforces the safety of this combination, even with long‐term follow‐up. Similarly, T‐DM1 and T‐DXd have been successfully combined with RT, though caution is warranted for CNS irradiation, where an increased risk of radiation necrosis has been observed. For HR+/HER2− breast cancer, CDK4/6i (palbociclib, ribociclib) have been safely administered concurrently with RT, with studies such as those by Beddok et al.^20^, ^21^ and Bouziane et al.^22^ demonstrating no significant increase in late toxicities. However, skin and hematologic toxicities remain a concern, requiring careful monitoring. For TNBC, pembrolizumab and RT appear to be well tolerated, with no major additional toxicity reported in early‐phase studies, though long‐term immune‐related complications, particularly myocarditis, require further evaluation. Olaparib, a PARP inhibitor, has also shown encouraging safety when combined with RT, as evidenced by the RADIOPARP trial,^29^ though additional research is needed to optimize dosing and patient selection. For metastatic settings, sacituzumab govitecan has been safely administered concurrently with RT, with no increase in RT‐induced toxicity observed in preliminary studies, supporting its feasibility for symptom control and disease management. While existing data strongly support the feasibility of combining novel systemic therapies with RT, prospective trials with extended follow‐up remain necessary to refine treatment sequencing, optimize patient selection, and establish definitive safety guidelines. The variability in RT techniques, fractionation schedules, and treatment volumes further underscores the need for standardized protocols to ensure consistent outcomes across clinical settings. Clinicians should continue to adopt a case‐by‐case approach, considering baseline cardiovascular risk, pulmonary function, immune‐related toxicity potential, and radiation field exposure. Multidisciplinary collaboration between radiation oncologists, medical oncologists, and cardiologists will be critical to maximizing treatment efficacy while minimizing risks.

The selection of RT techniques (Table 2) plays a crucial role in ensuring the safe and effective combination of modern systemic therapies with RT. As many targeted therapies, including HER2‐directed agents, CDK4/6i, immunotherapies, and antibody‐drug conjugates, are associated with cardiac, pulmonary, and hematologic toxicities, optimizing radiation delivery is essential to minimize treatment‐related side effects while maintaining oncologic efficacy. Advanced RT techniques such as proton therapy, isocentric lateral decubitus positioning, VMAT with DIBH, and HT offer effective strategies for reducing radiation exposure to critical organs. Given the growing integration of cardiotoxic systemic therapies with RT, the IC‐BRG recommends a personalized approach to RT planning, prioritizing proton therapy or lateral decubitus positioning when available. If these are not accessible, VMAT^9^ with DIBH^10^ should be the default approach, particularly for left‐sided breast cancer requiring nodal irradiation. The use of IMRT‐HT should be considered for patients requiring highly conformal dose distribution. As RT continues to evolve, standardized treatment protocols incorporating these advanced techniques will be essential to safely integrate novel systemic therapies, ensuring optimal tumor control while minimizing long‐term toxicities. Further prospective studies are needed to refine patient selection criteria and establish the best‐practice approaches for integrating modern systemic therapies with RT.

**TABLE 2:** Proposition of treatment techniques when combining radiotherapy with modern systemic treatments for breast cancer.

Indication	Gold‐standard technique	Comment
Breast only radiotherapy (with or without tumor bed boost)	Isocentric lateral decubitus	Improved left ventricle sparing compared with dorsal decubitus. In addition, lower dose bath exposure compared with VMAT (with or without DIBH) which may reduce the risk of auto‐reactive clone reactivation in case of pre‐existing immune myocarditis
Locoregional irradiation	VMAT	Standard technique, should be combined with DIBH for left‐sided breast cancer
	Helical tomotherapy	For complex anatomy (such as pectus excavatum)
	Protontherapy	For younger patient or radiosensibility/susceptibility mutations (ATM, TP53)

Abbreviations: DIBH, deep inspiration breath hold; VMAT, volumetric modulated arc therapy.

Several important limitations must be acknowledged in this expert opinion. The majority of evidence supporting concurrent systemic therapy and RT combinations derives from retrospective, observational studies, with most series being single‐institutional experiences that carry inherent selection bias and lack standardized protocols, while randomized controlled trials specifically designed to evaluate concurrent versus sequential approaches are largely absent from the literature. Many studies report short‐term follow‐up periods, particularly for newer agents such as T‐DXd and sacituzumab govitecan, which limits assessment of late toxicities, and sample sizes in several studies are small, reducing statistical power to detect rare but clinically significant adverse events. The predominance of single‐center data, particularly from Institut Curie, may limit generalizability to other institutions with different patient populations, treatment protocols, or RT techniques, and advanced RT techniques such as proton therapy and specialized positioning methods may not be universally available, potentially limiting the applicability of cardiac‐sparing recommendations. This work represents a narrative review combined with expert opinion rather than a systematic review or meta‐analysis, with the integration of published literature and institutional experience potentially introducing bias in recommendation formulation, while publication bias may favor positive results, potentially overestimating the safety of concurrent combinations. Significant variability exists across studies in RT techniques, fractionation schedules, treatment volumes, and systemic therapy dosing, making direct comparisons challenging and limiting the ability to establish standardized protocols, and limited long‐term follow‐up data are available for most combinations, particularly regarding late cardiac, pulmonary, and secondary malignancy risks, which may not manifest for years after treatment completion. These limitations underscore the need for prospective, randomized trials with standardized protocols and longer follow‐up to validate the safety and efficacy of concurrent systemic therapy and RT combinations in breast cancer management.

## 12 CONCLUSION

In summary, the concurrent use of RT with modern systemic therapies represents a viable and increasingly utilized strategy in breast cancer management. The evolving landscape of targeted agents offers unprecedented opportunities for treatment synergy, potentially improving both locoregional control and systemic disease outcomes. To ensure the safe and effective integration of these therapies, the use of advanced RT techniques is paramount. Further high‐quality prospective studies are essential to validate these promising findings and refine clinical decision‐making in the years to come.

## AUTHOR CONTRIBUTIONS

**Cezara Cheptea:** Writing – original draft; writing – review and editing. **Pierre Loap:** Writing – original draft; writing – review and editing. **Sofiane Allali:** Validation. **Alain Fourquet:** Validation. **Kim Cao:** Validation. **Youlia Kirova:** Validation.

## CONFLICT OF INTEREST STATEMENT

The authors declare no conflicts of interest.
